# Sensitivity of non-small cell lung cancer to erlotinib is regulated by the Notch/miR-223/*FBXW7* pathway

**DOI:** 10.1042/BSR20160478

**Published:** 2017-06-21

**Authors:** Haiwei Zhang, Fanglin Chen, Yongpeng He, Lin Yi, Chuang Ge, Xiaolong Shi, Chao Tang, Donglin Wang, Yongzhong Wu, Weiqi Nian

**Affiliations:** 1 Key Laboratory of Oncology, Chongqing cancer Hospital & Institute & Cancer center, Chongqing 400030, P.R. China; 2 Chongqing Key Laboratory of Translational Research for Cancer Metastasis and Individualized Treatment, Chongqing Cancer Institute, Chongqing 400030, P.R. China; 3 Cancer Institute of People’s Liberation Army, Xinqiao Hospital, Third Military Medical University, Chongqing 400037, P.R. China; 4 Department of Oncology, Chongqing Cancer Institute, Chongqing 400030, P.R. China; 5 Department of Radiotherapy, Chongqing Cancer Institute, Chongqing 400030, P.R. China

**Keywords:** Cell cycle, Cell apoptosis, Erlotinib, MicroRNA-223, Non-small cell lung cancer

## Abstract

Recent evidence supports a role for microRNA-223 (miR-223) in modulating tumor cell sensitivity to chemotherapeutic drugs; however, its role in cellular resistance to the effects of epidermal growth factor receptor tyrosine kinase inhibitors (EGFR-TKIs) used in treatment of non-small cell lung cancer (NSCLC) remains to be elucidated. The levels of miR-223 in parental cell line (HCC827) and erlotinib resistant HCC827 cell line (HCC827/ER) were detected by qRT-PCR. HCC827/ER cells were treated with MK-2206 to block the Akt signaling pathway or RO4929097 to block the Notch signaling pathway, and then transfected with an miR-223 inhibitor or interference expression plasmid of F-Box/WD repeat-containing protein 7 (FBXW7) or insulin-like growth factor 1 receptor (IGF1R). HCC827 cells were transfected with miR-223 mimics. Next, CCK-8, colony formation, and flow cytometric apoptosis assays were used to assess cell resistance to erlotinib. When compared with its expression in HCC827 cells, miR-223 expression was significantly up-regulated in HCC827/ER cells. Blocking either the Akt or Notch signaling pathway and reducing miR-223 expression resulted in decreased resistance in HCC827/ER cells. Conversely, increasing miR-223 expression induced cell resistance to erlotinib in HCC827 cells. miR-223 enhanced resistance to erlotinib by down-regulating *FBXW7* expression. Reducing *FBXW7* expression lowered resistance to erlotinib in HCC827/ER cells, while interference with expression of *IGF1R* produced no significant effect. This study demonstrated that NSCLC cells can up-regulate their levels of miR-223 expression via the Akt and Notch signaling pathways. miR-223 may serve as an important regulator of erlotinib sensitivity in NSCLC cells by targeting *FBXW7*.

## Introduction

Lung cancer is the most common type of cancer worldwide, and almost 80% of lung cancers are classified as non-small cell lung cancer (NSCLC) [1,2]. NSCLC may remain the leading cause of cancer death during the next 50 years, despite numerous therapies that have been developed for its treatment, including surgical procedures, chemotherapeutic agents, and forms of radiotherapy [3]. The Food and Drug Administration (FDA) has approved a series of epidermal growth factor receptor tyrosine kinase inhibitors (EGFR-TKIs), including gefitinib (Iressa®) and erlotinib (Tarceva®), which have remarkably increased the 5-year survival rate of cancer patients with relatively high levels of EGFR expression or some form of EGFR mutation [4–6]. Resistance to EGFR-TKIs usually develops over time and makes them unable to control cancer; however, the mechanisms that enable this drug resistance remain unknown. Numerous signaling pathways, such as the PI3K/Akt, Notch-1, JAK/STAT, Wnt, and TGF-β, have been shown to play key roles in NSCLC resistance to chemotherapeutic agents [7–12]. Among these resistance-associated cellular signal transduction pathways, the PI3K/Akt and Notch-1 cascades are well documented to mediate cancer resistance [13].

Because multidrug resistance (MDR) is a difficult problem to overcome when seeking to cure a cancer patient, numerous mechanisms for MDR have been investigated. For example, recent progress has been made in developing therapies that target cancer stem cells (CSCs), as high levels of CSCs are thought to be associated with tumor recurrence and chemotherapy resistance [14]. Many studies have suggested that CD44^+^ cell numbers are more representative of CSC numbers than are CD44^−^ cell numbers [15–17]. Thus the CD44^+^ phenotype is now acknowledged to be a biomarker for CSCs, and is widely used in the sorting and purification of CSCs.

Recent studies have suggested that microRNAs (miRNAs) capable of altering expression of their target genes are also associated with NSCLC resistance to EGFR-TKIs [18]. For example, several studies have shown that miRNA-223 (miR-223) acts as an onco-miRNA in several types of cancers [19–30]. Furthermore, related studies indicate that miR-223 reverses drug resistance by inhibiting the insulin-like growth factor 1 receptor (IGF1R)/P13K/Akt signaling pathway or production of ATP-binding cassette subfamily B member 1 (ABCB1) [7,31].

In our study, we found that miR-223 levels were up-regulated in erlotinib resistant HCC827 cell line (HCC827/ER) when compared with their levels in parental cell line (HCC827). Our study also showed that *IGF1R* and *FBXW7* were target genes for miR-223. We hypothesize that overexpression of miR-223 may down-regulate *FBXW7* expression, resulting in erlotinib resistance in NSCLC tumors. Here, we provide evidence supporting our hypothesis.

## Materials and methods

### Cell lines and reagents

Human NSCLC cells HCC827 (Cat no. TCHu73) and human embryonic kidney 293T cells (Cat no. SCSP-502) were obtained from the Cell Bank of the Chinese Academy of Science (Shanghai, China). The erlotinib resistant HCC827 cell line was defined as HCC827/ER cells. HCC827/ER cells with acquired resistance to erlotinib were obtained from the Key Laboratory of Oncology, Chongqing Cancer Institute. The HCC827 and 293T cells were cultured in DMEM (HyClone, Cat no. SH30243.01B) supplemented with 10% FBS (BI Biotech, Cat no. 04-001-1A). The HCC827/ER cells were maintained in 10% FBS DMEM supplemented with 1–5 µM erlotinib. All cells were cultured at 37°C in a humidified incubator containing 5% CO_2_. Erlotinib (Cat no. S7786), MK-2206 (Cat no. S1078), and RO4929097 (Cat no. S1575) were obtained from Selleck Chemicals; (Houston, TX, U.S.A.). To prevent the effects of erlotinib, the HCC827/ER cells were cultured in a normal medium for ≥2 weeks before their use in further experiments.

### Cell toxicity assay

HCC827 cells either pretreated with MK-2206 (an Akt inhibitor), RO4929097 (a Notch inhibitor) or transfected with miR-223 mimics, NC-siRNA lentiviruses, F-Box/WD repeat-containing protein 7 (FBXW7)-siRNA lentiviruses, or IGF1R-siRNA lentiviruses were treated with serially diluted concentrations of erlotinib (0, 0.1, 0.5, 1, 2, 5, or 10 µM) for 24 h. HCC827/ER cells transfected with an miR-223 inhibitor, empty vector or *FBXW7* plasmid were treated with serially diluted concentrations of erlotinib (5, 15, 25, 35, 45, or 55 μM) for 24 h. After treatment, 10 µl of CCK-8 solution was added to each well, and the incubations were continued for another 1–2 h. The optical density of each well at 450 nm (OD_450_) was detected using a New Epoch™ 2 Epoch Microplate Spectrophotometer (Biotek; Winooski, VT, U.S.A.).

### Dual-luciferase reporter assay

The plasmids of firefly luciferase reporter FBXW7/IGF1R-WT (wild-type miR-223-binding site in the 3′-UTR of IGF1R/FBXW7) and FBXW7/IGF1R-MUT (mutated miR-223-binding site in the 3′-UTR of IGF1R/FBXW7) were constructed by Genechem (Shanghai Genechem Co., Ltd; Shanghai, China). The miR-223 mimic and negative control (NC) plasmids were obtained from RiboBio (Guangzhou RiboBio Co., Ltd; Guangzhou, China). The firefly luciferase reporter (0.05 µg), miR-223 mimic, NC, and 0.01 µg of Renilla luciferase (an internal reference vector) were co-transfected into 293T cells using Lipofectamine™ 2000. Luciferase activity (fluorescence intensity) was measured with a fluorophotometer at 36 h after transfection.

### Lentivirus-mediated siRNA knockdown of *FBXW7* and *IGF1R*

The siRNA sequence targeting the *FBXW7* gene (NM_001013415.1) was 5′-CAAACTGTGATGAAGATATTT-3′; the siRNA sequence targeting the *IGF1R* gene (NM_000875.4) was 5′-GGAAACTCTTCTACAACTACG-3′. The NC siRNA was 5′-TGCGCTGCT GGTGCCAACCCTATTCT-3′. The respective products were cloned into pcDNA3.1 (Invitrogen; Carlsbad, CA, U.S.A.). The constructed vectors and lentivirus packaging vectors (pMD2.G, pMDL-G/P-RRE, and pRSV-REV) were co-transfected into 293T cells for 48 h respectively. Lentivirus particles were harvested and purified by ultracentrifugation. HCC827 cells (10,000 cells/well) were seeded into 24-well plates and transfected with lentivirus using 8 μg/ml polybrene (Sigma; St. Louis, MO, U.S.A.). Cells showing stable expression were isolated by filtration into medium containing 800 μg/ml G418 (Sigma).

### Construction of the *FBXW7* overexpression vector

The full-length *FBXW7* coding sequence was amplified from cDNA that was synthesized by the reverse transcriptase polymerase chain reaction (RT-PCR) using the total 5 ug of RNA extracted from HCC827 cells as a template. Briefly, cDNA was synthesized by reverse transcription using the oligo dT_18_, and then used as a template for PCR amplification of the full-length *FBXW7* coding sequence. The forward primer sequence for *FBXW7* was: 5′-ATTTGCGGCCGCATGAATCAGGAACTGCTCTCT-3′ and the reverse primer sequence was 5′-CCGCTCGAGTCACTTCATGTCCACATCAAA-3′. The PCR products were purified using a gel extraction purification kit (OMEGA Bio-Tek; Norcross, GA, U.S.A.); after which, they were digested with BamHI and NotI and inserted into the pcDNA3.1 vector (Invitrogen) to obtain a recombinant plasmid (*FBXW7*/pcDNA3.1), which was subsequently sent to Sangon Company (Shanghai, China) for sequencing. HCC827/ER cells were seeded into a 24-well plates (10,000 cells/well) and transfected with pcDNA3.1 or *FBXW7*/pcDNA3.1 using Lipofectamine™ 2000. Cells showing stable expression were isolated by filtration and stored in medium containing 800 μg/ml G418 (Sigma).

### Quantitative real-time PCR

Total cellular RNA was purified from HCC827 and HCC827/ER cells using TRIzol® reagent (Invitrogen, Cat no. 15596-018). RNA quantity and quality were determined by spectrophotometry and agarose gel electrophoresis respectively. A 1 μg sample of extracted RNA was reverse transcribed into cDNA using a Bestar^TM^ qPCR RT Kit following the manufacturer’s protocol with a slight modification. Quantitative RT-PCR was performed with DBI Bestar® SYBRGreen qPCR Master Mix according to the manufacturer’s instructions describing its use with an Agilent Stratagene QRT-PCR Mx3000P Detection System (Agilent; Santa Clara, CA, U.S.A.). The primer sequences are shown in Table 1, and the cycling conditions were as follows: initial denaturation at 95°C for 60 s, followed by 40 cycles of 95°C for 5 s and 58°C for 20 s. Data were analyzed using the 2^−ΔΔ*C*^ method.

**Table 1:** Primers for quantitative RT-PCR

	Sequence 5′–3′
hsa-miR-223-5p	CGTGTATTTGACAAGCTGAGTT
miR-223-5p-RT	CTCAACTGGTGTCGTGGAGTCGGCAATTCAGTTGAGAACTCAGC
miR-223-5p-F	ACACTCCAGCTGGGCGTGTATTTGAC
miR-223-5p-R	CTCAACTGGTGTCGTGGA
U6 F	CTCGCTTCGGCAGCACA
U6 R	AACGCTTCACGAATTTGCGT
GAPDH F	ACACCCACTCCTCCACCTTT
GAPDH R	TTACTCCTTGGAGGCCATGT
FBXW7 F	CGTGTTTGGGATGTGGAGAC
FBXW7 R	TGATGCTTGTTGGGACCTTG
IGFR F	CGCCTCCAACTTCGTCTTT
IGFR R	CCTCAACTTGTGATCCGTATTTT

### Western blot studies

Cells were harvested and lysed in ice-cold lysis buffer (50 mM Tris, 2% SDS, 5% glycerinum, 100 mM NaCl, and 1 mM EDTA, pH 6.8). The total protein content of the lysate was measured using a BCA Protein Assay Kit (Pierce Biotechnology, Cat no. 23235). Equal amounts of protein (30 μg) were separated by SDS/10% PAGE; after which, the protein bands were transferred onto a PVDF membrane (BioRad, Cat no. 162-0177), which was then incubated with rabbit anti-FBXW7 (Proteintech, Cat no. 117515-1-AP; 1:1000 dilution), rabbit anti-IGF1R (Cell Signaling Technology, Cat no. 4668; 1:1000 dilution), rabbit anti-p-AKT (Cell Signaling Technology, Cat no. 9252; 1:1000 dilution), rabbit anti-AKT (Cell Signaling Technology, Cat no. 9215; 1:500 dilution), rabbit anti-Notch (Cell Signaling Technology, Cat no. 9212; 1:1000 dilution), and rabbit anti-GAPDH (Proteintech, Cat no. 10494-1-AP; 1:1000 dilution) antibodies overnight at 4°C. The membrane was then incubated with horseradish-peroxidase (HRP)-conjugated goat anti-rabbit IgG antibody (Santa Cruz Biotechnology, Cat no. SC-2054; 1:5000 dilution). The signal intensity of each protein band was measured with an ECL-PLUS/Kit (Amersham, Cat no. RPN2132) according to the manufacturer’s protocol. GAPDH served as an internal control standard. The relative density of bands were quantified by quantity one (Bio-Rad).

### Colony formation assay

Cells were seeded into six-well plates (800 cells/well) and incubated for 9 days to form colonies of suitable size. The cell medium was replaced every 3 days. Next, the cells were incubated with paraformaldehyde for 30 min at 25°C; after which, the fixed cells were washed twice with PBS, stained for 10 min with 1% Crystal Violet (Beyotime, Cat no. C0121), washed with ddH_2_O, and dried in air. The total number of colonies containing >50 cells was counted under a light microscope. Image data analysis was performed using Image-Pro Plus 6.0 software (Media Cybernetics; Rockville, MD, U.S.A.).

### Flow cytometry

The numbers of CSCs (DD44^+^ phenotype) and percentages of apoptotic cells were assayed by flow cytometry. Briefly, the cells were trypsinized, collected, and washed. Next, cells to be analyzed for the CD44^+^ phenotype were stained with anti-CD44 antibodies (Sigma–Aldrich) for 30 min at 4°C according to the manufacturer’s protocol (Sigma). Cells to be analyzed for apoptosis were stained with Annexin V-FITC (BD Biosciences; Franklin Lakes, NJ, U.S.A.) and PI in the dark for 10 min at 4°C according to the manufacturer’s protocol (BD Biosciences).

### Bioinformatics and statistical analysis

The predicted targets of miR-223 were predicted through three different algorithms: TargetScan v7.1 (http://www.targetscan.org/), miRanda (http://www.microrna.org/microrna/home.), and miRDB (http://www.mirdb.org/miRDB/). Data analysis was performed using GraphPad Prism 5.01 software (GraphPad Software, Inc; La Jolla, CA, U.S.A.). All assays were repeated at least three times, and results are presented as the mean ± SD. One-way ANOVA was used to determine the statistical significance of differences between groups, and a *P*-value <0.05 was considered to be statistically significant.

## Results

### Overexpression of miR-223 in HCC827/ER cells

**Figure 1:**
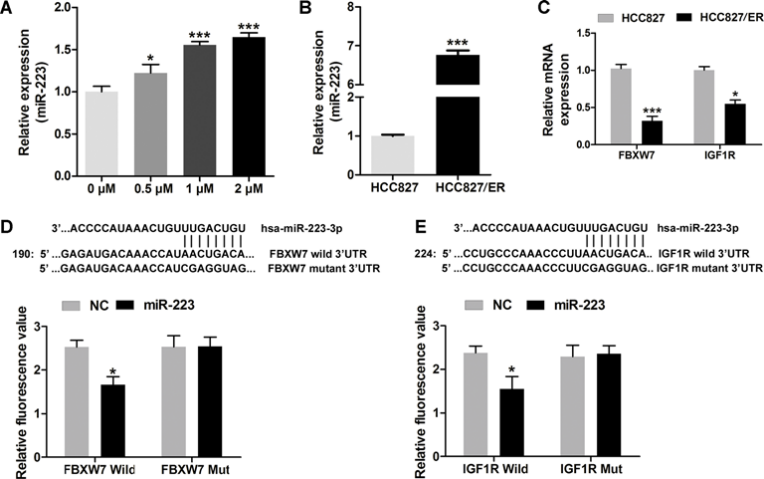
Down-regulated FBXW7 and IGF1R may induced by overexpression of miR-223 in HCC827/ER cells. Overexpression of miR-223 in HCC827/ER cells. (**A**) The levels of miR-223 in HCC827 cells during their treatment with serially diluted erlotinib (0, 0.1, 0.5, 1, and 2 µM) for 48 h as analyzed by q-PCR. (**B**) The levels of miR-223 in HCC827 and HCC827/ER cells as analyzed by q-PCR. (**C** and **D**) The binding sites and target genes (*IGF1R* and *FBXW7*) for miR-223 as predicted by Targetscan. Wild-type (FBXW7/IGF1R 3’-UTR-WT) or mutant (FBXW7/IGF1R 3′-UTR-MUT) reporter plasmids were co-transfected along with miR-223 or an NC into 293T cells. Normalized luciferase activity in the control group was used for calculating relative luciferase activity. (**E**) IGF1R and FBXW7 mRNA levels in HCC827 and HCC827/ER cells as analyzed by q-PCR; *P<0.05 and ***P<0.001.

To evaluate the role of miR-223 during chemotherapy (erlotinib) of NSCLC, HCC827 cells were treated with serial dilutions of erlotinib (0, 0.1, 0.5, 1, or 2 µM) for 48 h; after which, their levels of miR-223 expression were analyzed by qRT-PCR. The results showed that miR-223 expression in the HCC827 cells was increased during erlotinib treatment (Figure 1A). To explore the role of miR-223 in NSCLC chemotherapy resistance, we analyzed the levels of miR-223 expression in HCC827 and HCC827/ER cells by qRT-PCR and then compared the two levels. We found higher levels of miR-223 expression in the HCC827/ER cells than in the HCC827 cells (Figure 1B), suggesting that miR-223 might play a key role in the acquired resistance to erlotinib in HCC827 cells. Our qRT-PCR data also showed that the levels of *FBXW7* and *IGF1R* expression in HCC827 cells were higher than those in HCC827/ER cells (Figure 1C). A bioinformatics analysis identified *FBXW7* and *IGF1R* (Figure 1D and E) as possible target genes for miR-223, and dual-luciferase reporter assays verified that prediction. The fluorescence signal in FBXW7/IGF1R Wild group was significantly lower than that of FBXW7/IGF1R Mut group when the cells were transfected with miR-223. These results led us to speculate that miR-223 may be involved in NSCLC resistance to erlotinib through regulating FBXW7/IGF1R.

### Inhibition of the Akt and Notch signaling pathways decreased miR-223 levels and reversed chemoresistance to erlotinib in HCC827/ER cells

**Figure 2:**
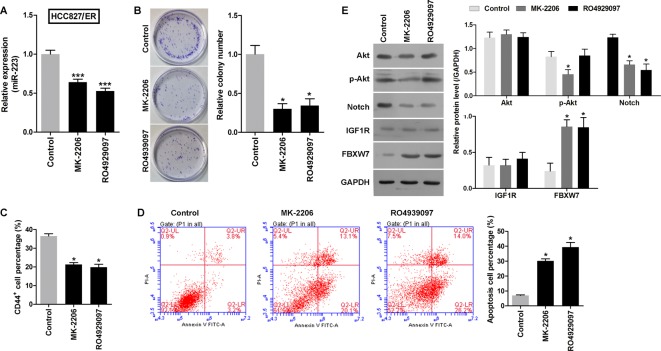
Akt and Notch signaling pathways and miR-223 in HCC827/ER cells. The levels of miR-223 in HCC827/ER cells were regulated by the Akt and Notch signaling pathways, and inhibition of those pathways reversed chemoresistance to erlotinib. (**A**) The levels of miR-223 in HCC827/ER cells during their treatment with MK-2206 (an Akt inhibitor) or RO4929097 (a Notch inhibitor) for 48 h, as analyzed by q-PCR. (**B**) Images recorded with a MicroView imager showing the number of colonies formed by each population of cells. (**C**) The percentage of CD44^+^ cells in each group of HCC827/ER cells during 48 h of treatment with MK-2206 (an Akt inhibitor) or RO4929097 (a Notch inhibitor). (**D**) Representative data from FACS analyses of cell apoptosis levels, and the percentage of apoptotic cells in each group of HCC827/ER cells during 48 h of treatment with MK-2206 (an Akt inhibitor) or RO4929097 (a Notch inhibitor). (**E**) Western blots showing expression of p-Akt, Akt, Notch, IGF1R, and *FBXW7* in HCC827/ER cells treated with MK-2206 (an Akt inhibitor) or RO4929097 (a Notch inhibitor). GAPDH served as an internal control. All data represent the mean value ± SD from three independent experiments; *P<0.05 and ***P<0.001.

Previous research has shown that the Notch and Akt pathways are abnormally active when NSCLC becomes resistant to chemotherapy [8,32]. Thus, we investigated a possible correlation between Notch/Akt activity and miR-223 expression in HCC827/ER cells. HCC827/ER cells were treated with MK-2206 (an Akt inhibitor) or RO4929097 (a Notch inhibitor) for 48 h; after which, their levels of miR-223 expression were analyzed by qRT-PCR. The data showed that miR-223 expression in HCC827/ER cells was decreased during treatment with either an Akt or Notch inhibitor (Figure 2A). This suggested that lower levels of miR-223 may enhance the sensitivity of HCC827/ER cells to erlotinib. The effects of an Akt inhibitor and Notch inhibitor on chemoresistance to erlotinib were also analyzed with the CCK-8 assay. As shown in Table 2, the IC_50_ values for the Akt inhibitor and Notch inhibitor in the pretreatment groups were lower than those in the control group. Furthermore, the colony numbers in the Akt inhibitor and Notch inhibitor pretreatment groups were also lower than those in the control group (Figure 2B). While the percentages of CSCs (CD44^+^ subpopulation) in the Akt inhibitor and Notch inhibitor pretreatment groups were lower than those in the control group (Figure 2C), the percentages of apoptotic cells in the Akt inhibitor and Notch inhibitor pretreatment groups were higher than those in the control group (Figure 2D). Additionally, the levels of *FBXW7* expression in the HCC827/ER cells increased when the Akt and Notch pathways were inhibited (Figure 2E). These results demonstrated that miR-223 levels in HCC827/ER cells were regulated by the Akt and Notch signaling pathways, and affected chemoresistance to erlotinib.

**Table 2:** Effects of miR-223, MK-2206, RO4929097, IGF1R, and FBXW7 on the IC_50_ of erlotinib in HCC827 or HCC827/ER cells

Cell type	Group	IC_50_ (µM)
HCC827	—	3.85 ± 0.41
HCC827/ER	—	29.68 ± 1.24*
	MK-2206	20.72 ± 2.11^†^
	RO4929097	21.45 ± 1.47^†^
HCC827	NC	3.88 ± 0.55
	miR-223 mimics	4.91 ± 0.59^‡^
HCC827/ER	NC	29.55 ± 2.08
	miR-223 inhibitor	20.645 ± 0.96^§^
HCC827	Vector	3.59 ± 0.87
	FBXW7-siRNA	8.77 ± 0.74^║^
HCC827	Vector	3.72 ± 0.29
	IGF1R-siRNA	3.70 ± 0.59
HCC827/ER	Vector	29.66 ± 1.87
	FBXW7	19.76 ± 1.21^¶^

All data represented as mean value ± SD from three independent experiments. “*”, significantly different from HCC827, *P*<0.05. “†”, significantly different from HCC827/ER Control group, *P*<0.05. “‡”, significantly different from HCC827 NC group, *P*<0.05. “§”, significantly different from HCC827/ER NC Vector group, *P*<0.05. “║”, significantly different from HCC827 NC Vector group, *P*<0.05. “¶”, significantly different from its HCC827/ER NC Vector group, *P*<0.05.

### Overexpression of miR-223 reduced sensitivity to erlotinib in HCC827 cells by down-regulating *FBXW7* expression

**Figure 3:**
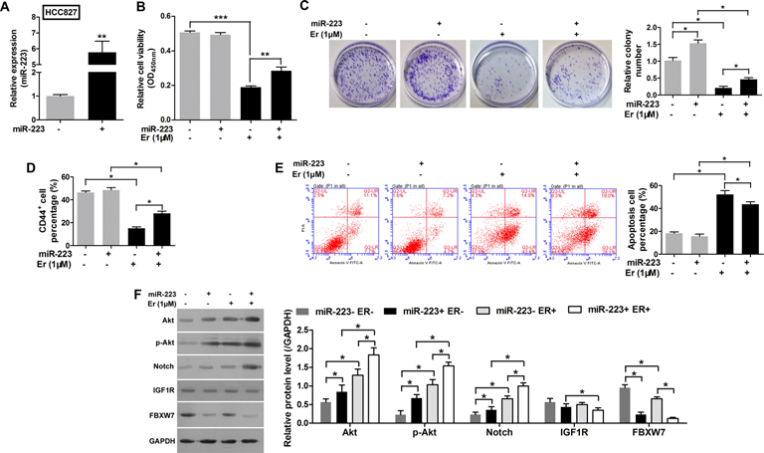
Overexpression of miR-223 reduced the sensitivity of HCC827 cells to erlotinib by enhancing the Notch pathway but not the AKT pathway. (**A**) The levels of miR-223 in HCC827 cells transfected with miR-223 mimics or an NC. (**B**) Cell viability as evaluated with the CCK-8 assay. The HCC827 cells were first transfected with miR-223 mimics and then treated with 1 µM erlotinib. (**C**) Images recorded with a MicroView imager showing the numbers of colonies formed by HCC827 cells transfected with miR-223 mimics and then treated with erlotinib. (**D**) The percentage of CD44^+^ cells in each group of HCC827 cells transfected with miR-223 mimics and then treated with erlotinib. (**E**) Representative data from FACS analyses of cell apoptosis levels, and the percentage of apoptotic cells among HCC827 cells transfected with miR-223 mimics and then treated with erlotinib. (**F**) Western blot analyze the expression of p-Akt, Akt, Notch, IGF1R, and FBXW7 in HCC827 cells transfected with miR-223 mimics and then treated with erlotinib. GAPDH served as an internal control. All data represent the mean value ± SD from three independent experiments; *P<0.05, **P<0.01, and ***P<0.001.

To investigate whether overexpression of miR-223 would alter cellular sensitivity to erlotinib, miR-223 mimic was successfully transfected into HCC827 cells (Figure 3A). Next, the HCC827 cells were transfected with mimic-miR-223 or mimic-NC to examine the effects of altered miR-223 expression on the antitumor activity of erlotinib in those cells. Although erlotinib exhibited antitumor activity in the HCC827 cells, overexpression of miR-223 had significantly reduced their sensitivity to erlotinib (Figure 3B and Table 2). Moreover, the HCC827 cell population transfected with miR-223 mimics displayed a greater colony formation ability (Figure 3C) and included a higher percentage of CD44^+^ subpopulation cells (Figure 3D). The miR-223 reversed the lower colony formation ability and increased the less CD44+ subpopulation cells induced by erlotinib (Figure 3C and D).The percentage of apoptosis HCC827 cells increased under treatment of erlotinib, but the miR-223 could down-regulate the percentage of apoptosis cells (Figure 3E). These results indicated that miR-223 induced resistance to erlotinib in HCC827 cells. We also found that *FBXW7* expression was decreased in miR-223 overexpressing cells and that Notch and Akt pathway activity levels in HCC827 cells were increased during erlotinib treatment (Figure 3F). The Notch and Akt pathway may be the targets of miR-223, which lead to the drug resistance to erlotinib in NSCLC.

### An miR-223 inhibitor partially reversed chemoresistance to erlotinib in HCC827/ER cells by enhancing *FBXW7* expression

**Figure 4:**
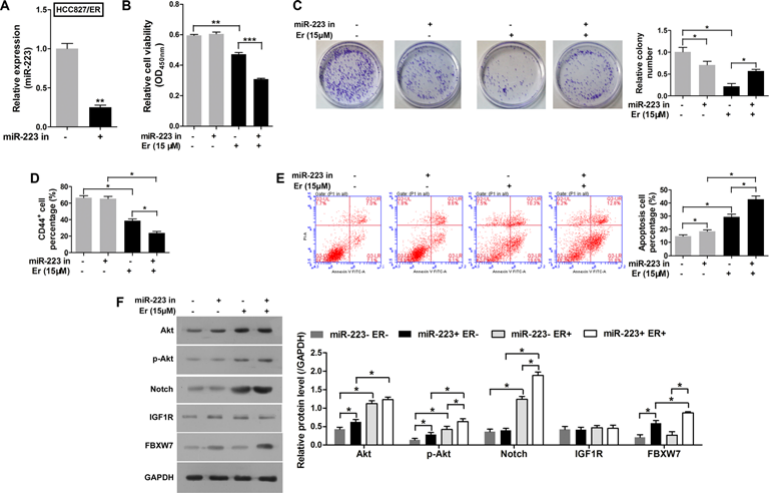
An miR-223 inhibitor partially reversed chemoresistance to erlotinib in HCC827/ER cells by enhancing the Notch pathway. (**A**) The levels of miR-223 in HCC827/ER cells transfected with an miR-223 inhibitor or NC. (**B**) Cell viability was evaluated with the CCK-8 assay. The HCC827/ER cells were first transfected with an miR-223 inhibitor and then treated with erlotinib. (**C**) Images recorded with a MicroView imager show the numbers of colonies formed by HCC827/ER cells transfected with an miR-223 inhibitor and then treated with erlotinib. (**D**) The percentage of CD44^+^ cells in each group of HCC827/ER cells transfected with the miR-233 inhibitor and then treated with erlotinib. (**E**) Representative data from FACS analyses of cell apoptosis, and the percentage of apoptotic cells among HCC827/ER cells transfected with the miR-223 inhibitor and then treated with erlotinib. (**F**) The HCC827/ER cells transfected with the miR-223 inhibitor and then treated with erlotinib were detected by Western blot to analyze the expression of p-Akt, Akt, Notch, IGF1R, and FBXW7. GAPDH served as an internal control. All data represent the mean value ± SD from three independent experiments; *P<0.05, **P<0.01 and ***P<0.001.

Conversely, to investigate whether suppression of miR-223 would alter cellular sensitivity to erlotinib, an miR-223 inhibitor was successfully transfected into HCC827/ER cells (Figure 4A); after which, CCK-8 assays were performed with both the transfected and normal control cells. Although the HCC827/ER cells still displayed resistance to erlotinib, suppression of miR-223 significantly decreased their level of resistance (Figure 4B and Table 2). Moreover, inhibition of miR-223 decreased the colony formation ability of the cells (Figure 4C). Their CD44^+^ subpopulation percentage (Figure 4D) were reduced, while the number of apoptotic cells were increased during erlotinib treatment. *FBXW7* expression was increased in cells that overexpressed the miR-223 inhibitor (Figure 4E). We also found higher levels of Notch activity in the HCC827/ER cells during erlotinib treatment (Figure 4F). These results indicate that the miR-223 inhibitor had reversed the resistance to erlotinib in HCC827/ER cells.

### *FBXW7* reduces HCC827 cells’ chemoresistance to erlotinib

**Figure 5:**
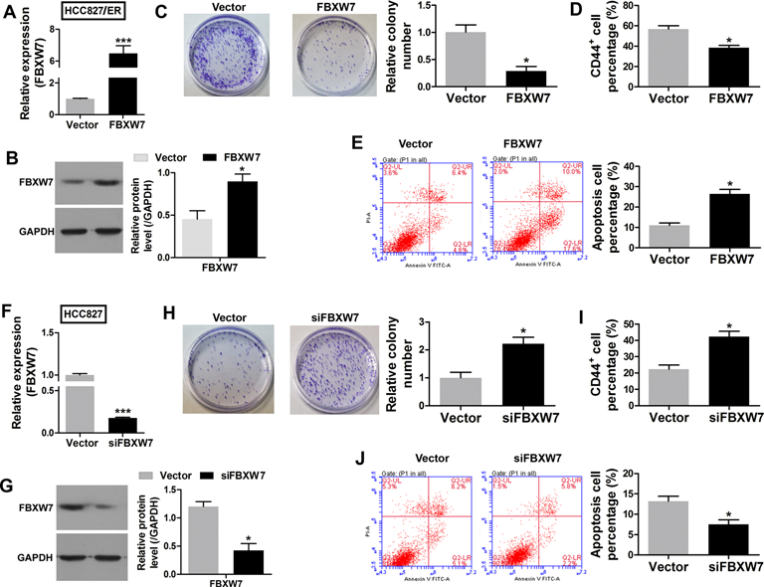
*FBXW7* suppressed chemoresistance to erlotinib. The *FBXW7* mRNA (**A**) and protein (**B**) levels in HCC827 cells transfected with *FBXW7*/pCDNA3.1 or an empty vector. (**C**) Images recorded with a MicroView imager show the numbers of colonies formed by HCC827 cells transfected with *FBXW7*/pCDNA3.1 or an empty vector. (**D**) The percentage of CD44^+^ in each group of HCC827 cells transfected with *FBXW7*/pCDNA3.0 or an empty vector. (**E**) Representative data from FACS analyses of cell apoptosis levels, and the percentage of apoptotic cells among HCC827 cells transfected with FBXW7/pCDNA3.0 or an empty vector. The *FBXW7* mRNA (**F**) and protein (**G**) levels in HCC827/ER cells transfected with *FBXW7* siRNA or empty vector lentivirus. (**H**) Images recorded with a MicroView imager showing the numbers of colonies formed by HCC827/ER cells transfected with *FBXW7* siRNA or empty vector lentivirus. (**I**) The percentage of CD44^+^ in each group of HCC827 cells transfected with FBXW7siRNA or empty vector lentivirus. (**J**) Representative data from FACS analyses of cell apoptosis levels, and the percentage of apoptotic cells among HCC827 cells transfected with FBXW7 siRNA or empty vector lentivirus. All data represent the mean value ± SD from three independent experiments; *P<0.05.

To investigate whether the miR-223 target gene (*FBXW7*) acts to suppress chemoresistance, a *FBXW7* overexpression vector was successfully transfected into HCC827/ER cells (Figure 5A and B). CCK-8 assays were then performed with the HCC827/ER cells transfected with the FBXW7 overexpression vector and cells transfected with an empty vector. Overexpression of *FBXW7* significantly enhanced the sensitivity of the transfected HCC827/ER cells to erlotinib (Table 2). Furthermore, overexpression of *FBXW7* reduced the colony formation ability of the cells (Figure 5C) and their CD44^+^ subpopulation percentage (Figure 5D), but increased the percentage of apoptotic cells (Figure 5E).

Conversely, high *FBXW7* mRNA levels in the HCC827 cells suggested a potential suppressor role for that gene in chemoresistance. Knockdown of *FBXW7* by lentivirus-mediated siRNA markedly lowered the *FBXW7* mRNA and protein levels in HCC827 cells (Figure 5F) and significantly increased the proliferation of those cells, as demonstrated by CCK-8 assay results (Table 2). The *FBXW7* siRNA-mediated growth suppressive effect was further confirmed by the results colony formation assays and examining the percentages of CD44^+^ subpopulation cells. A significant reduction in colony numbers (Figure 5H) and the percentage of CD44^+^ subpopulation cells (Figure 5I), accompanied by a decrease in the numbers of apoptotic cells (Figure 5J) was observed among cells transfected with *FBXW7* siRNA when compared with cells transfected with an empty vector.

## Discussion

The development of tumor resistance is a complex multifactorial process involving the secondary mutation of target genes, activation of alternative pathways, ROS, aberrant expression of microRNAs, ATP-binding cassette (ABC) transporter effusion, and histologic transformations that must occur to allow resistance to EGFR-TKIs [33]. Identification of the molecules and metabolic pathways that contribute to resistance is crucial for understanding chemoresistance and developing alternative therapeutic strategies. Recent evidence has shown that miR-223 serves as an onco-miRNA during development of chemotherapy resistance and cancer metastasis [4,8,34–36]; however, its role in resistance to EGFR-TKIs remains controversial [7,31]. Our results suggest that miRNA-223 (miR-223) acts as an onco-miRNA with resistance effect to EGFR-TKIs. We used dual-luciferase reporter assays to prove that miR-223 directly targets *FBXW7* and *IGF1R* mRNA by binding to its target sequence in each mRNA molecule. Our study also verified that inhibitors of the Akt and Notch pathways could reverse resistance to erlotinib in HCC827/ER cells by reducing miR-223 levels. Furthermore, suppression of miR-223 with Akt and Notch inhibitors enhanced the sensitivity of HCC827/ER cells to erlotinib by increasing *FBXW7* expression. These pathways may play roles in the development of resistance to erlotinib in NSCLC cells. Li et al. [36] found that continuous activation of the PI3K/Akt signaling pathway could enhance cellular resistance to gefitinib and erlotinib, and that a blocker of PI3K/Akt signaling increased the sensitivity of cancer cells to both of those agents by inducing apoptosis in both *in vitro* and *in vivo* models. Notch-1 contributes to acquisition of the epithelial–mesenchymal transition (EMT) phenotype, which is key factor connected with acquired resistance to gefitinib. Suppression of Notch-1 activity with siRNA or an inhibitor induces mesenchymal–epithelial transition, which is associated with impaired metastasis, cell invasion, cell growth, and drug resistance [8,37–40]. The CD44+ was used as a biomarker of CSC that represents the tumorigenic ability [14–17]. MK-2206 or RO4929097 decreased the CD44+ cell percentage in HCC827/ER, which indicated that the tumorigenic ability could be inhibited by MK-2206 or RO4929097. In HCC827 cells, the CD44+ cell percentage was down-regulated by erlotinib but reversed by miR-223, which indicated miR-223 promotes the resistance of HCC827 cells to erlotinib.

*FBXW7*, which is regulated by miR-223, has been identified as a tumor suppressor gene in several cancers [21,35,41,42], and plays key roles in modulating the degradation of various onco-protein substrates, including c-Myc, cyclinE, Notch, c-Jun, mTOR, and MCL1 [43]. A recent study by Inuzuka and Wertz et al. [44,45] revealed that *FBXW7* plays a pivotal role in regulating the EGF and HER2 signaling pathways, and regulates the apoptotic pathway by inhibiting MCL1 degradation. Our results also show that the miR-223/FBXW7 pathway may play an important role in modulating the sensitivity of NSCLC cells to erlotinib. IGFs and the IGF1R are possibly involved in the cellular response to Herceptin, as they may activate the Akt/Bad and mTOR signaling pathways in breast carcinoma cells [46,47]. Moreover, Lee et al. [48,49] reported that *IGF1R* is involved in resistance to EGFR-TKIs.

When investigating whether the targeting of *IGF1R* by miR-223 contributes to chemoresistance, we found that knockdown of *IGF1R* by lentivirus-mediated siRNA markedly lowered the *IGF1R* mRNA and protein levels (Supplementary Figure S1A), but had no effect on HCC827 cell proliferation as detected with CCK-8 assays (Table 2). The colony formation ability (Supplementary Figure S1B) and percentages of CD44^+^ subpopulation cells (Supplementary Figure S1C) and apoptotic cells (Supplementary Figure S1D) were nearly identical. On the contrary, our results showed that *IGF1R* was not associated with the sensitivity of HCC827 cells to erlotinib.

In conclusion, our study is the first to demonstrate that miR-223 is overexpressed and continuously activates the Akt and Notch signaling pathways in HCC827/ER cells. miR-223 may be a key onco-miRNA modulating the sensitivity of NSCLC cells to erlotinib by inhibiting *FBXW7* but not *IGF1R*. Moreover, the Notch/miR-223/FBXW7 pathway represents a promising therapeutic target for NSCLC patients whose tumors are resistant to erlotinib. Although further studies in patient-derived tumor xenograft (PDX) animal models will be required to confirm that this mechanism is responsible for acquired resistance to erlotinib, our results can be used to facilitate the development of new treatments for NSCLC patients who do not respond erlotinib.

## Abbreviations

ABC: ATP-binding cassette
ABCB1: ATP-binding cassette subfamily B member 1
CSC: cancer stem cell
DMEM: Dulbecco's modified eagle medium
EGFR-TKI: epidermal growth factor receptor tyrosine kinase inhibitor
EMT: epithelial–mesenchymal transition
FBXW7: F-Box/WD repeat-containing protein 7
HRP: horseradish-peroxidase
GAPDH: glyceraldehyde-3-phosphate dehydrogenase
IGF1R: insulin-like growth factor 1 receptor
JAK: Janus kinase
MDR: multidrug resistance
mTOR: mechanistic target of rapamycin
NC: negative control
NSCLC: non-small cell lung cancer
PI3K: phosphatidylinostiol 3-kinase
QRT-PCR: quantitative reverse transcriptase polymerase chain reaction
STAT: signal transducers and activators of transcription
TGF-beta: transforming growth factor-beta
